# Improving the production of acetyl-CoA-derived chemicals in *Escherichia coli* BL21(DE3) through *iclR* and *arcA* deletion

**DOI:** 10.1186/s12866-016-0913-2

**Published:** 2017-01-07

**Authors:** Min Liu, Yamei Ding, Hailin Chen, Zhe Zhao, Huizhou Liu, Mo Xian, Guang Zhao

**Affiliations:** 1 CAS Key Laboratory of Biobased Materials, Qingdao Institute of Bioenergy and Bioprocess Technology, Chinese Academy of Sciences, Qingdao, 266101 China; 2 University of Chinese Academy of Sciences, Beijing, 100049 China; 3 Institute of Oceanology, Chinese Academy of Sciences, Qingdao, 266071 China; 4 Randian Technology Company Limited, Tianjin, 300457 China

**Keywords:** Acetyl-CoA, *Escherichia coli*, *iclR*, *arcA*, 3-hydroxypropionate, Phloroglucinol

## Abstract

**Background:**

Acetyl-CoA-derived chemicals are suitable for multiple applications in many industries. The bio-production of these chemicals has become imperative owing to the economic and environmental problems. However, acetate overflow is the major drawback for acetyl-CoA-derived chemicals production. Approaches for overcoming acetate overflow may be beneficial for the production of acetyl-CoA-derived chemicals.

**Results:**

In this study, a transcriptional regulator *iclR* was knocked out in *E.coli* BL21(DE3) to overcome acetate overflow and improve the chemicals production. Two important acetyl-CoA-derived chemicals, phloroglucinol (PG) and 3-hydroxypropionate (3HP) were used to evaluate it. It is revealed that knockout of *iclR* significantly increased expressions of *aceBAK* operon. The cell yields and glucose utilization efficiencies were higher than those of control strains. The acetate concentrations were decreased by more than 50% and the productions of PG and 3HP were increased more than twice in *iclR* mutants. The effects of *iclR* knockout on cell physiology, cell metabolism and production of acetyl-CoA-derived chemicals were similar to those of *arcA* knockout in our previous study. However, the *arcA-iclR* double mutants couldn’t gain higher productions of PG and 3HP. The mechanisms are unclear and needed to be resolved in future.

**Conclusions:**

Knockout of *iclR* significantly increased gene expression of *aceBAK* operon and concomitantly activated glyoxylate pathway. This genetic modification may be a good way to overcome acetate overflow, and improve the production of a wide range of acetyl-CoA-derived chemicals.

## Background

**Fig. 1:**
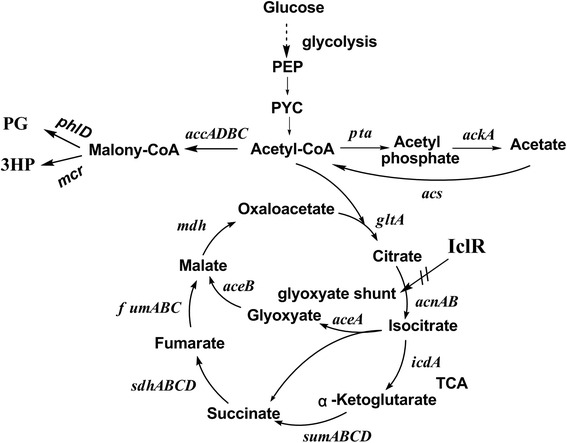
The main carbon metabolic pathways in recombinant *E.coli* strains

Acetyl-CoA-derived chemicals, including polyhydroxyalkanoates, isoprenoids, polyketides, lipids and butyrate, are suitable for multiple applications in food additive, medicine, agriculture, cosmetic, and chemical industries [1–3]. The bio-production of acetyl-CoA-derived chemicals has become imperative owing to the diminishing petroleum reserves and growing environmental concerns [4]. To date, *Escherichia coli* remains one of the most widely used hosts in recombinant bioprocesses because of its low manufacturing cost and easy manipulation [5, 6]. The bio-production of acetyl-CoA-derived chemicals through engineering *Escherichia coli* has made significant progresses in recent years. However, it is worth mentioning that acetyl-CoA can be converted to acetate through the acetate kinase/phosphate acetyl transferase (AckA-Pta) pathway when cells grow rapidly on glucose abundant conditions, also known as ‘acetate overflow’ [7–9] (Fig. 1). Acetate excretion affects the cell density, biomass accumulation and macromolecule biosynthesis even at concentrations as low as 0.5 g/l [10, 11]. Mutations in both *ackA* and *pta* have been reported a strong reduction of acetate production. However, this is at the expense of the cell growth rate and is accompanied by an increase in the production of other by-products such as formate and lactate [12]. So, decreasing the undesirable conversion from acetyl-CoA to acetate may be beneficial for acetyl-CoA-derived chemicals production. As predicted, our previous study has revealed that knockout of *arcA* in *E.coli* BL21(DE3) exhibited surprising efficacy of overcoming acetate formation, and the productions of acetyl-CoA-derived chemicals PG and 3HP were improved by 2.25-fold and 2.08-fold, respectively [13].

Acetate overflow is observed in all *Escherichia coli* strains but the extent can differ greatly between the various strains [7, 14]. *E.coli* BL21 strains showed lower acetate yields as compared to K12 strains [15, 16]. C^13^-Constrained metabolic flux analysis revealed that the lower acetate yields in BL21 were caused by the higher activation of glyoxylate pathway and acetate assimilation pathway [15, 17]. IclR (isocitrate lyase regulator) is a local regulator, repressing the gene expression of *aceBAK* operon, which codes for the glyoxylate pathway enzymes malate synthase (AceB), isocitrate lyase (AceA) and isocitrate dehydrogenase kinase/phosphatase(AceK) [18–20] (Fig. 1). Decreasing the gene expression of *iclR* may activate glyoxylate pathway and decrease acetate formation. If not, lots of acetyl-CoA would be transformed to acetate through AckA-Pta pathway, which in turn starve the precursor for the acetyl-CoA-derived chemicals production and hurt the yields.

In this study, the *iclR* gene of *E.coli* BL21(DE3) was knocked out, and the resultant strain was used to produce two acetyl-CoA-derived chemicals, phloroglucinol (PG) and 3-hydroxypropionate (3HP) which are both important bulk chemicals [21, 22]. The effects of *iclR* deletion on gene expression of *aceBAK* operon, the cell physiology and metabolism were also determined and discussed. As *arcA* deletion also had positive effects on PG and 3HP production, the cell physiology and metabolism and production of acetyl-CoA-derived chemicals in *E.coli* BL21(DE3) *arcA*-*iclR* double mutant were also investigated in this study.

## Methods

### Strains construction

The oligonucleotide primers used in this study were listed in Table 1, and the recombinant plasmids and strains used in this study were listed in Table 2. The *iclR* mutants were constructed using P1 phage transduction from BL21(DE3) and *arcA* single mutant Q1949 as previously described [23]. The donor strain of *E.coli* BW25113 *iclR*::*kan* (JW3978) was purchased from the Keio collection [24]. The kanamycin genes were removed by a temperature-sensitive plasmid of pCP20, and the resultant strains were named as Q2280 and Q2138, respectively . The four subunits of *accA*, *accD* and *accBC* from *E.coli* were cloned into pACYCDuet-1, resulting in plasmid pA-*accADBC.* The *phlD* gene from *P. fluorescens* Pf5 (ATCC BAA -477) and *mar* gene from *E.coli* were cloned into pET30a, resulting in plasmid pET-*phlDmar.* [13, 22] The plasmids pA-*accADBC* and pET-*phlDmar* were transformed into *E.coli* BL21(DE3), *arcA* mutant Q1949, *iclR* mutant Q2280 and *arcA*-*iclR* double mutant Q2138 to generate PG-producing strains Q1944, Q1963, Q2283 and Q2176. The *mcr*-N and *mcr-*C (N_940_V/ K_1106_W/ S_1114_R) from *Chloroflexus aurantiacus* were cloned into pETDuet-1, resulting in plasmid pMCR-N-C [25]. The plasmids pA-*accADBC* and pMCR-N-C were also transformed into *E.coli* BL21(DE3), Q1949, Q2280 and Q2138 to generate 3HP -producing strains Q2204, Q2284, Q2285 and Q2286.

**Table 1:** Primers used in this study

Primer name	Sequence
Strain construction	
ID-*iclR*_F	TACGAAATGCCGGATCGTTG
ID-*iclR*_R	TCTTGTTTATCAAGAGTGTC
pKD13-Kan_R	GGTGAGATGACAGGAGATCC
RT-PCR Reaction	
16S rRNA_F	TGGTCTGAGAGGATGACCAG
16S rRNA_R	TGCTTCTTCTGCGGGTAACG
*aceB*_F	ATGACTGAACAGGCAACAAC
*aceB*_R	CGAATGGAAGCT GTTTCCGA
*aceA*_F	ATGAAAACCCGTACACAACA
*aceA*_R	CGAGGCTGTTGATGTAGCCT
*aceK*_F	CGTGGCCTGGAATTATTGAT
*aceK*_R	GTTAGTAATGCAGCGCAGTT

**Table 2:** Plasmids and strains used in this study

Plasmids and strains	Description	Source
Plasmids		
pETDuet-1	Amp^*r*^ *oripBR322 lacI* ^*q*^ *T*_*7*_ *p*	Novagen
pACYCDuet-1	Cm^*r*^ *oriP15A lacI* ^*q*^ *T*_*7*_ *p*	Novagen
pCP20	Cm^r^, Amp^r^ pSC101 ori *CI857*	Novagen
pA-*accADBC*	rep_p15A_ Cm^R^ *lacI* P_T7_ *accA* P_T7_ *accD* P_T7_ *accBC*	[22]
pET-*phlDmar*	rep_pBR322_ kan^R^ *lacI* P_T7_ *phlD* P_T7_ *mar*	[22]
pMCR-N-C	rep_pBR322_ Amp^R^ *lacI* P_T7_ *His*_*6*_ *-mcr*_1-549_ P_T7_ *His*_*6*_ *-mcr*_550-1219_(N_940_V/ K_1106_W/ S_1114_R)	[25]
Strains		
*E. coli* DH5α	F^-^ *supE*44 Δ*lacU*169 (*ϕ*80 *lacZ* Δ*M15*) *hsdR*17 *recA*1 *endA*1 *gyrA*96 *thi*-1 *relA*1	Invitrogen
*E. coli* BL21(DE3)	F^-^ *ompT gal dcm lon hsdSB* (rB^-^ mB^-^) λ(DE3)	Invitrogen
JW3978	*E. coli* BW25113 *iclR*::*kan*	Keio collection
Q1949	*E. coli* BL21(DE3)*△arcA*	[13]
Q2133	*E. coli* BL21(DE3) *iclR*::*kan*	This study
Q2280	*E. coli* BL21(DE3)*△iclR*	This study
Q2135	*E. coli* BL21(DE3)*△arcA iclR*::*kan*	This study
Q2138	*E. coli* BL21(DE3)*△arcA△iclR*	This study
Q1944	*E. coli* BL21(DE3)/pA-*accADBC/*pET-*phlDmar*	This study
Q1963	Q1949/pA-*accADBC/*pET-*phlDmar*	This study
Q2283	Q2280/pA-*accADBC/*pET-*phlDmar*	This study
Q2176	Q2138/pA-*accADBC/*pET-*phlDmar*	This study
Q2204	*E. coli* BL21(DE3)/pA-*accADBC/*pMCR-N-C	This study
Q2284	Q1949/pA-*accADBC/*pMCR-N-C	This study
Q2285	Q2280/pA-*accADBC/*pMCR-N-C	This study
Q2286	Q2138/pA-*accADBC/*pMCR-N-C	This study

### Gene expression by RT-PCR

Total RNAs from BL21(DE3) and Q2280 were isolated using Bacteria RNA Kit (omega) according to the manufacturer’s recommendations. The quantity and purity of RNAs were determined by optical density measurements at 260nm and 280nm by 1% agarose gel electrophoresis. Reverse-transcription (RT)-PCR reactions were carried out in a PCR thermocycler using TransScrip® One-step gDNA removal and cDNA synthesis kit. The reaction mix was first incubated 10min at 25°C and then incubated 30 min at 42°C for reverse transcription. Inactivated gDNA remover by heating 5 s at 85°C. Gene expression levels of *aceBAK* operon in BL21(DE3) and *iclR* mutant Q2280 were analyzed by PCR amplification with primers in Table 1. The gene transcription levels of 16S rRNA were used as inner reference for normalization.

### Shake-flask cultivation of the recombinant *E.coli* strains

Shake-flask experiments were carried out in triplicate in 250 ml flask containing 50 ml fermentation medium. The strains were grown overnight at 37°C with shaking in LB broth, and then 1:50 diluted into 50 ml fermentation medium. When OD_600_ of the culture reached about 0.6, IPTG was added to a final concentration of 0.1 mM/l and further incubated at 30°C. 10 mg/l Biotin and 20mM/l NaHCO_3_ were needed for 3HP production. The fermentation medium for PG production contains 9.8 g/l K_2_HPO_4_•3H_2_O, 2.1 g/l citric acid•H_2_O, 0.3 g/l ferric ammonium citrate, 3.0 g/l (NH_4_)_2_SO_4_, 0.2 g/l MgSO_4_•7H_2_O, 20 g/l glucose and 1000 × trace mental (3.7 g/l (NH_4_)_6_Mo_7_O_24_•4H_2_O, 2.9 g/l ZnSO_4_•7H_2_O, 24.7 g/l H_3_BO_3_, 2.5 g/l CuSO_4_•5H_2_O, 15.8 g/l MnCl_2_•4H_2_O). The fermentation medium for 3HP production contains 14 g/l K_2_HPO4•3H_2_O, 5.2 g/l KH_2_PO_4_, 1 g/l NaCl, 1 g/l NH_4_Cl, 0.25 g/l MgSO_4_•7H_2_O, 0.2 g/l yeast extract, 20 g/l glucose. The OD_600_ of the culture and the concentrations of residual glucose were measured during the whole fermentation course.

### Analytical methods

The cell concentration was measured by the optical density of the culture at 600 nm, and the value of density was converted to cell dry weight (CDW) based on that one unit of OD_600_ was equivalent to 0.36 g/l CDW. The concentration of residual glucose was quantified by using an SBA-40D Biological Sensing Analyzer. The concentration of PG in the fermentation supernatant was quantified using the colorimetric reaction at OD_446_ between cinnamaldehyde and PG. [26] The concentrations of 3HP and acetate in medium were determined by HPLC as described previously [13, 27].

## Results

### Knockout of *iclR* increased gene expression of *aceBAK* operon

**Fig. 2:**
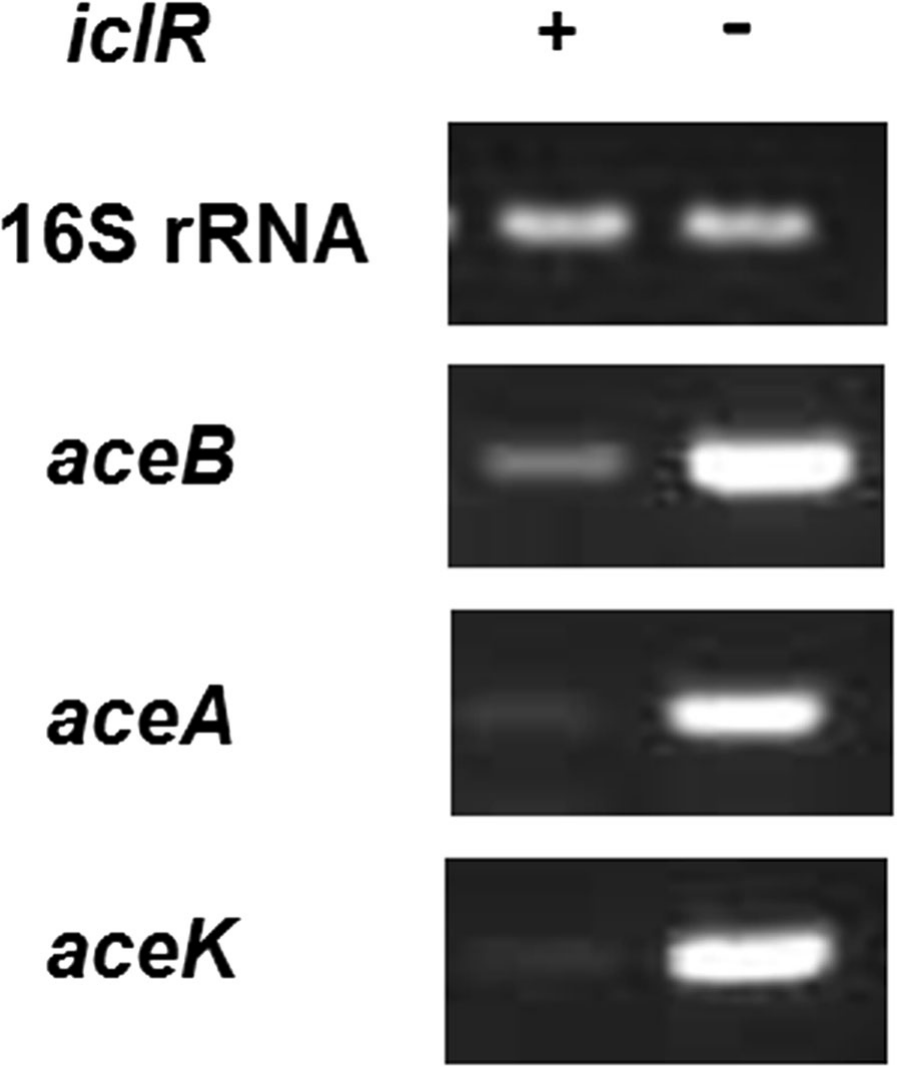
Genes transcription of *aceBAK* operon in *E.coli* BL21(DE3) and *iclR* mutant by RT-PCR. Constitutively, gene transcriptional level of 16S rRNA was used as inner reference

IclR is a transcriptional regulator that regulates gene expression of *aceBAK* operon [18, 20]. In order to investigate the effect of *iclR* deletion on gene expression of *aceBAK* operon, the total RNAs of wild strain BL21(DE3) and *iclR* mutant strain Q2280 were extracted to carry out the RT-PCR reaction. The gene transcription levels of 16S rRNA were used as inner reference for normalization. As shown in Fig. 2, the mRNA levels of *aceB*, *aceA* and *aceK* in *iclR* mutant Q2280 were much higher than those of the wild-type strain BL21(DE3), while the mRNA levels of 16S rRNA were very similar in these two strains, suggesting that *iclR* deletion could up-regulate the gene expression of *aceBAK* operon.

### Effects of *iclR* knockout on cell physiology and metabolism

**Fig. 3:**
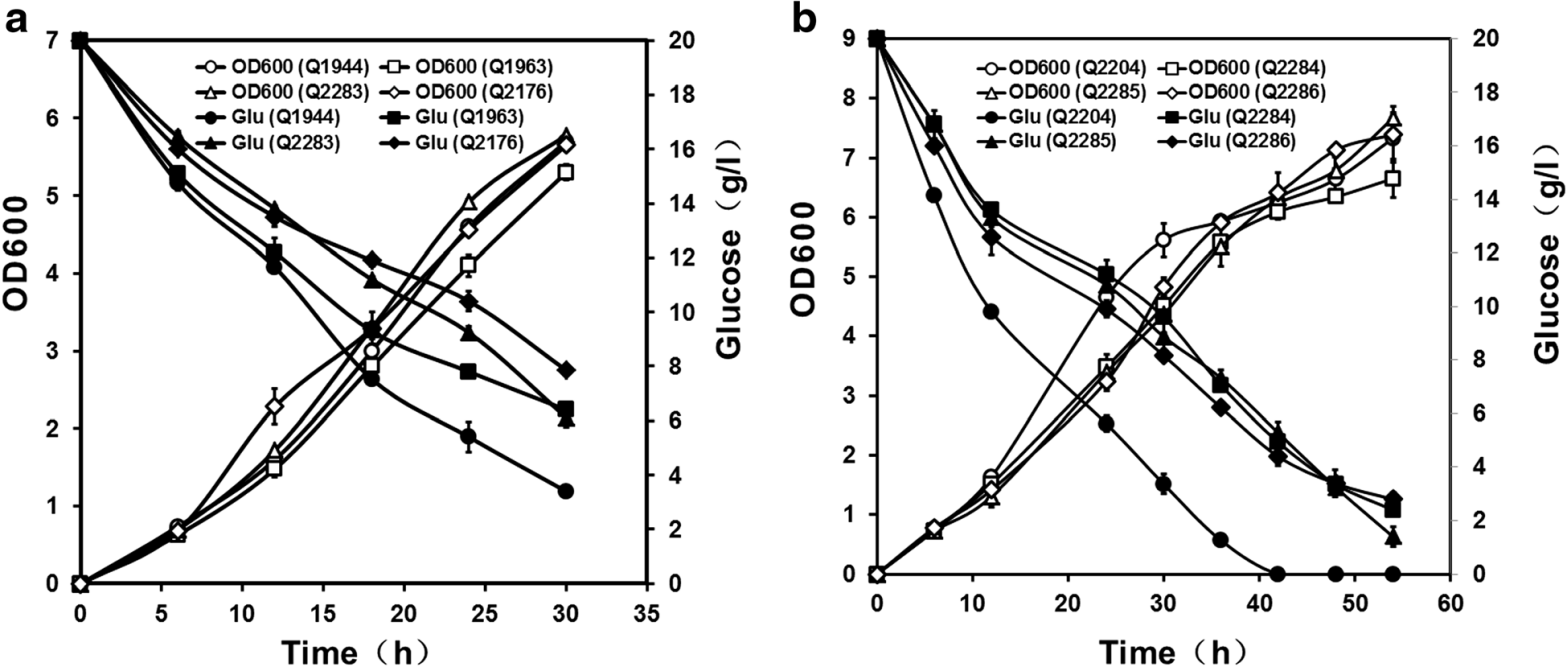
Cell growth and glucose consumption of recombinant *E.coli* strains during the process of cultivation in shaking flasks. **a** The OD600 and residual glucose concentrations of PG-producing strains Q1944, Q1963, Q2283 and Q2176 in their cultures. **b** The OD600 and residual glucose concentrations of 3HP-producing strains Q2204, Q2284, Q2285 and Q2286 in their cultures

**Fig. 4:**
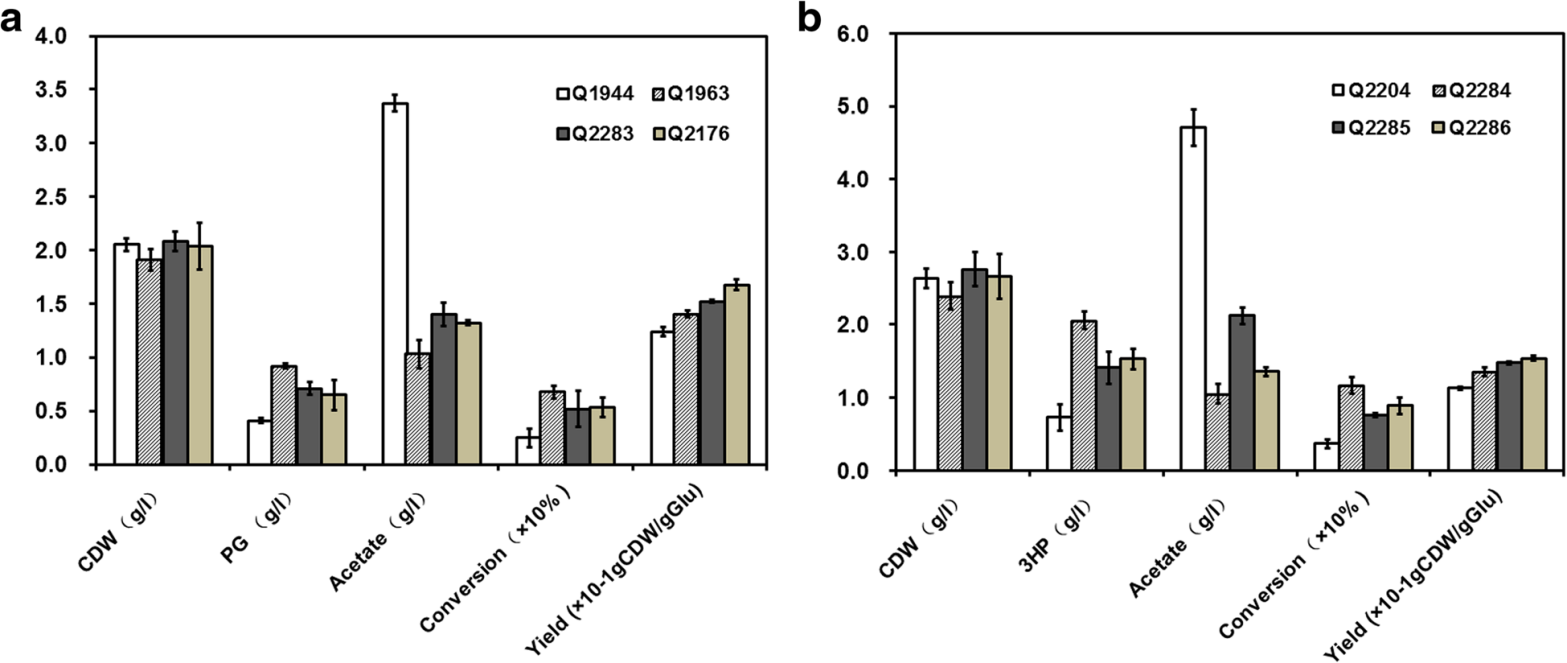
The effects of *arcA* and *iclR* knockout on the cell phycology and metabolism of recombinant *E.coli* strains during the process of cultivation in shaking flasks. **a** The cell dry weight, PG productions, acetate concentrations, glucose conversion efficiencies and cell yields of PG-producing strains. **b** The cell dry weight, 3HP productions, acetate concentrations, glucose conversion efficiencies and cell yields of 3HP-producing strains

The *iclR* mutants were constructed using P1 phage transduction and the recombinant plasmids were transformed for PG and 3HP production. During the cultivation process, the OD_600_ and residual glucose concentrations of the control strains (Q1944 and Q2204) and *iclR* mutants (Q2283 and Q2285) were measured to investigate the effects of *iclR* deletion on cell growth and glucose utilization. As shown in Fig. 3 and Fig. 4, the *iclR* mutants showed similar cell dry weight (CDW) as compared to their corresponding control strains. After fermentation, the cell dry weight of PG-producing strains Q1944 and Q2283 were 2.05 ± 0.06 g/l and 2.08 ± 0.09 g/l, while 3HP-producing strains Q2204 and Q2285 accumulated 2.64 ± 0.13 g/l and 2.76 ± 0.22 g/l CDW, respectively. These results drew a conclusion that deletion of *iclR* had no obvious effect on cell growth during the bio-production of PG and 3HP. Meanwhile, the glucose consumptions and cell yields were also compared. The glucose consumptions of *iclR* mutants obviously decreased since IPTG induction. Finally, the residual glucose concentrations of *iclR* mutants Q2283 and Q2285 were significantly higher than their corresponding control strains (Fig. 3). Knockout of *iclR*

decreased the glucose consumptions without impairing the cell dry weight, therefore, *iclR* mutants possessed higher cell yields. It is speculated that the genetic modification of *iclR* knockout could improve the glucose utilization efficiency and reduce the manufacturing cost in industrial applications.

Acetate is the major undesirable metabolite in the production of acetyl-CoA-derived chemicals. After cultivation, the control strain Q1944 and Q2204 produced 3.14 ± 0.13 g/l and 5.86 ± 0.05 g/l acetate, while the *iclR* mutants Q2283 and Q2285 produced 1.41 ± 0.01 g/l and 2.12 ± 0.13 g/l acetate, decreased by 55% and 64%, respectively. In conclusion, *iclR* deletion showed dramatic effects on glucose utilization efficiency and acetate formation during the process of PG and 3HP production. However, the cell growth was very similar between the control strains and *iclR* mutants.

### Knockout of *iclR* improved PG and 3HP production

The effects of *iclR* deletion on PG and 3HP production were also investigated (Fig. 4). The PG production of *iclR* mutant Q2283 was 0.71 ± 0.06 g/l, showing 1.7-time higher than that of the control strain Q1944. The glucose conversion efficiency to PG was increased by 2.1-fold, from 2.47% to 5.19%. In 3HP production, the *iclR* mutant Q2285 produced 1.41 ± 0.22 g/l 3HP, showing 1.9-time higher than that of the control strain Q2204. The glucose conversion efficiency to 3HP was increased by 2.0-fold, from 3.65% to 7.58%. Knockout of *iclR* significantly improved the production of acetyl-CoA-derived chemicals and the glucose conversion efficiency.

### Effects of *arcA* and *iclR* knockouts on cell physiology and metabolism

Summarizing the above results, deletion of *arcA* or *iclR* can improve the cell yield and glucose utilization efficiency and repress the acetate formation, so we are curious whether the double knockout *of arcA* and *iclR* should further affect the cell physiology and metabolism. The detailed comparison of the cell growth, glucose consumption, cell yield and acetate production in *arcA* mutants, *iclR* mutants and *arcA-iclR* double mutants were also made. As shown in Fig. 3 and Fig. 4, the cell growth was seriously impaired through *arcA* deletion. While, it was not affected in *iclR* mutants and *arcA-iclR* double mutants. The double mutants Q2176 and Q2286 produced 2.03 ± 0.22 g/l and 2.66 ± 0.31 g/l CDW, the same as the control strains of 2.05 ± 0.06 g/l and 2.64 ± 0.12 g/l CDW. In addition, the cell yields of *arcA-iclR* double mutants were slightly higher than those of the control strains, the *arcA* single mutants and the *iclR* single mutants. It was assumed that the combined deletion of *arcA* and *iclR* was beneficial for the conversion from glucose to the biomass.

All the mutant strains showed reduced acetate productions as compared to the control strains (Fig. 4). However, the ability to overcome acetate formation of *arcA-iclR* double mutants was less than that of the *arcA* single mutants. The regulatory mechanisms remained unclear and needed to be resolved.

### Effects of *arcA* and *iclR* knockouts on PG and 3HP production

The *arcA-iclR* double mutants showed lower productions of PG and 3HP than the *arcA* single mutants. The productions of PG and 3HP in *arcA* single mutants were increased by 2.25-fold and 2.83-fold than those in the control strains Q1944 and Q2204, while they were only increased by 1.58-fold and 2.15-fold in *arcA-iclR* double mutants. Consistent with that effects, the *arcA-iclR* double mutants also showed lower glucose conversion efficiencies than the *arcA* single mutants. The glucose conversions efficiencies to PG and 3HP in *arcA* single mutants were 6.76% and 11.7% respectively, while they were only 5.35% and 8.89% in *arcA-iclR* double mutants.

Taken together, both mutants of *arcA* and *iclR* showed positive effects on the productions and glucose conversion efficiencies to PG and 3HP. However the *arcA* single mutants had the most prominent results, and the *iclR* single mutants, the *arcA-iclR* double mutants showed the similar effects on these aspects.

## Discussion

The excretion and assimilation of acetate usually undergo a metabolic switch when cells growing on glucose. Acetate is produced by the Pta-AckA pathway in exponential growth phase, and it is converted to acetyl-CoA by the catalysis of acetyl-CoA synthetase (Acs) with the cells convert to stationary phase [28]. As the intracellular enzyme pyroposphatase removes the intermediate pyrophosphate, the reaction from acetate to acetyl-CoA is irreversible. Acetyl-CoA was utilized to generate ATP and metabolic intermediates for the cellular activities and exogenous genes expression via TCA cycle and glyoxylate shunt. So, acetate overflow is not only detrimental to cell growth and macromolecular biosynthesis, it is also a loss of carbon and therefore an economic sink [12]. To date, numerous approaches have been reported to overcome acetate excretion. If some of these approaches could also be used to improve the production of target compounds and decrease the production cost, these studies will have important significance and market value. In this study, the effects of *arcA* and/or *iclR* deletion on the production of acetyl-CoA-derived chemicals were determined and discussed. The results revealed that the *arcA* and/or *iclR* deletion significantly decreased the acetate concentrations. All the mutants showed higher cell yields and higher glucose utilization efficiencies. More importantly, the productions of PG and 3HP were increased than those of the control strains (Fig. 3 and Fig. 4). So, overcoming acetate overflow through *arcA* and/or *iclR* deletion could improve the production of acetyl-CoA-derived chemicals. Acetate overflow is observed in all *Escherichia coli* strains, however, the extent is very different between the K-12 and B strains, which are the most common *E.coli* strains for laboratorial and biotechnological applications [6]. BL21 strains showed less acetate accumulations than K-12 strains during the process of high cell-density fermentation with glucose as the sole carbon. A comparison of the genome sequences between BL21 and K12 strains showed that BL21 strains possessed rare codons in *arcA* and two mutations in the promoter region of *iclR* [29]. These variations of BL21 resulted in the reduced expressions of *arcA* and *iclR*, presumably contributing to the lower acetate accumulation. A previous research revealed that the central metabolic fluxes of BL21(DE3), especially with respect to the TCA fluxes, the glyoxylate pathway and the pentose pathway were similar to that of the K12 strains with *arcA* and *iclR* deletion [29]. Meanwhile, the acetate concentration of *E. coli* MG1655 Δ *arcA* Δ *iclR* was decreased to the value of BL21(DE3) [30]. Considering all that, the expression levels of *arcA* and *iclR* in BL21 strains were lower than K12 strains due to the variations in rare codons and promoter region, respectively. A previous study considered that the levels of *iclR* gene and its gene products in *E. coli* BL21 strains were much lower compared to in K12 strains, probably, the *iclR* levels were so low that a complete deletion of *iclR* [14]. However, our results revealed that knockout of *iclR* in BL21(DE3) could also significantly decrease acetate producitons by more than 50% and improve the PG and 3HP productions by more than twice. Thus, genetic modifications of *iclR* in K12 and BL21 strains both had obvious effect on overcoming acetate overflow, though the *iclR* levels were already very low in BL21 strains.

ArcA (anaerobic redox control) is the cytosolic response regulator of the dual-component regulator system ArcAB [31, 32]. It is reported that phosphorylated ArcA can repress genes expression of TCA cycle and glyoxylate shunt, such as *gltA*, *acnAB*, *icdA*, *sucABCD*, *sdhCDAB*, *fumA*, *mdh,* and *aceB* [33–35]. Our previous results demonstrated that the acetate concentrations of *arcA* mutants in BL21(DE3) were decreased by more than 70% and the productions of PG and 3HP were increased by more than twice. The decreased acetate concentrations and increased PG and 3HP productions of *arcA* mutants could be caused by the following reasons: the higher activity of TCA cycle and glyoxylate pathway; the more metabolic flux through pyruvate dehydrogenase (PDH) complex, which catalyzes pyruvate to acetyl-CoA [13, 32, 36]. IclR (isocitrate lyase regulator) is a local regulator, inhibiting genes expression of *aceBAK* operon in glyoxylate pathway. Knockout of *iclR* significantly increased gene expression of *aceBAK* operon according to the results of RT-PCR (Fig. 2). In glyoxylate pathway, isocitrate is directly split into succinate and glyoxylate without CO_2_ loss, it may be used to explain the fact that *iclR* mutants showed higher cell yields than their corresponding control strains. Glyoxylate pathway is the main pathway for acetate assimilation, knockout of *iclR* can activate the glyoxylate pathway and promote the conversion from acetate to acetyl-CoA [17, 37]. In addtion, the metabolic flux from phosphoenolpyruvic acid (PEP) to acetyl-CoA is obviously increased in *iclR* mutant strains [29]. So, knockout of *iclR* can elevate the metabolic flux from acetate to acetyl-CoA and from PEP to acetyl-CoA. On the one hand, acetyl-CoA is metabolized to provide the energy and precursors for exogenous genes expression in recombinant pathways. And on the other hand, acetyl-CoA is the direct substrate for the production of acetyl-CoA-derived chemicals. Therefore, the *iclR* knockout can decrease the acetate formation and increase the production of acetyl-CoA-derived chemicals.

To summarize, the *arcA* deletion and *iclR* deletion were observed to behave similar effects on the cell physiology and metabolism, except the cell growth. The cell growth of *arcA* mutants was much lower than their corresponding control strains, however it was not impaired in *iclR* mutants. It still remains unclear what causes this phenomenon. Then, the *arcA* and *iclR* double mutants were also constructed to study the effects on the cell physiology, the metabolism and the production of acetyl-CoA-derived chemicals. Surprisingly, the double mutants had no better results for PG and 3HP productions than those of *arcA* and *iclR* single mutants. Meanwhile, the glucose conversion efficiencies and the abilities for overcoming acetate formation were also lower than those of the *arcA* single mutants. These results proved that the effects of polygenic modification on the produciton of target compounds may not be better than the single genic modification, especially by the global regulators. A global regulator usually controls several operons that belong to different groups [36]. Engineering of a global regulator could show pleiotropic phenotypes and variations in metabolic pathways and carbon fluxes. It is hard to determine the certain gene or the single pathway with a global regulator was modified. Furthmore, the results will be more unpredictable and complex when the several regulators are combined modified. In this study, why the effects of *arcA-iclR* double mutant on overcoming acetate overflow and improving the acetyl-CoA-derived chemicals production were less than the *arcA* single mutants? A previous study reported that *arcA-iclR* deletion of *E. coli* K12 MG1655 had dramatic effects on the cell physiology and metabolism. However, no significant differences of metabolic flux were observed between the wild type BL21(DE3) and the *arcA-iclR* double mutant [14]. The tiny variations of metabolic flux in BL21(DE3) *arcA-iclR* double mutant may lead to the less effects on the cell physiology and metabolism. However, the exact mechanisms would be unravelled by determining the distribution of metabolic fluxes in *arcA* single mutant, *iclR* single mutant and *arcA-iclR* double mutant in future.

## Conclusions

Acetate overflow is the major drawback for acetyl-CoA-derived chemicals production. Approaches for overcoming acetate overflow may be beneficial for the production of acetyl-CoA-derived chemicals. IclR is a transcriptional regulator that regulates gene expression of *aceBAK* operon in glyoxylate pathway. In this work, *iclR* was knocked out in *E. coli* BL21(DE3) to construct a mutant strain, and the productions of two important acetyl-CoA-derived chemicals, phloroglucinol and 3-hydroxypropionate were used to evaluate it. The results revealed that knockout of *iclR* significantly improved gene expression of *aceBAK* operon. The *iclR* mutants showed higher cell yields and higher glucose utilization efficiencies without sacrificing cell growth. The acetate concentrations were decreased by more than 50%. More importantly, the productions of PG and 3HP were 2-times higher than those of control strains. These phenomena of *iclR* knockout were similar to those of the global regulator *arcA* knockout in our previous study. However, the *arcA-iclR* double mutants could not produce higher productions of PG and 3HP. Considering all that, the genetic modification of *iclR* would be a good choice to improve the production of a wide range of acetyl-CoA-derived chemicals in industry.

## Abbreviations

3HP: 3-hydroxypropionate
Ace: Acetate
DCW: Dry cell weight
Glu: Glucose
PEP: Phosphoenolpyruvate
PG: Phloroglucinol
RT-PCR: Reverse-transcription polymerase chain reaction
SDS-PAGE: Sodium dodecyl sulfate-polyacrylamide gel electrophoresis
